# Effects of Negative Pressure Wound Therapy on Levels of Angiopoetin-2 and Other Selected Circulating Signaling Molecules in Patients with Diabetic Foot Ulcer

**DOI:** 10.1155/2019/1756798

**Published:** 2019-10-28

**Authors:** Jerzy Hohendorff, Anna Drozdz, Sebastian Borys, Agnieszka H. Ludwig-Slomczynska, Beata Kiec-Wilk, Ewa L. Stepien, Maciej T. Malecki

**Affiliations:** ^1^Department of Metabolic Diseases, Jagiellonian University Medical College, Krakow, Poland; ^2^Clinic of Metabolic Diseases, University Hospital, Krakow, Poland; ^3^Department of Medical Physics, Marian Smoluchowski Institute of Physics, Jagiellonian University, Krakow, Poland; ^4^Malopolska Center of Biotechnology, Jagiellonian University, Krakow, Poland; ^5^Center for Medical Genomics OMICRON, Jagiellonian University Medical College, Krakow, Poland

## Abstract

**Background and Aims:**

Diabetic foot ulcers (DFUs) are linked to amputations and premature deaths. Negative pressure wound therapy (NPWT) has been used for DFUs. The mechanism of NPWT's action may be associated with its influence on circulating molecules. We assessed NPWT's effect on the plasma levels of angiopoietin-2 (Ang2), a key regulator of angiogenesis, and its microvesicular receptors (Tie2) as well as the microvesicles (MVs) themselves in DFU patients.

**Materials and Methods:**

We included 69 patients with type 2 diabetes mellitus (T2DM) and neuropathic, noninfected DFUs—49 were treated with NPWT and 20 were treated with standard therapy (ST). Assigning patients to the NPWT group was not random but based on DFU characteristics, especially wound area. Ang2 was measured by ELISA in the entire group, while in a subgroup of 19 individuals on NPWT and 10 on ST, flow cytometry was used to measure Tie2+ and the corresponding isotype control (Iso+) and annexin V (AnnV+) as well as total MVs. Measurements were performed at the beginning and after 8 ± 1 days of therapy.

**Results:**

Treatment groups were similar for basic characteristics but differed by their median DFU areas (10.3 (4.2-18.9) vs. 1.3 (0.9-3.4) cm^2^, *p* = 0.0001). At day 0, no difference was observed in Ang2 levels, total MVs, MV Tie+, and MV AnnV+ between the groups. Ang2 decreased after 8 days in the NPWT group, unlike in the ST group (3.54 (2.40-5.40) vs. 3.32 (2.33-4.61), *p* = 0.02, and 3.19 ± 1.11 vs. 3.19 ± 1.29 ng/mL, *p* = 0.98, respectively). No other parameters were identified that may have been influenced by the NPWT treatment.

**Conclusion:**

NPWT in T2DM patients with neuropathic, noninfected DFU seems to lead to reduction of the Ang2 level. Influencing the level of Ang2 may constitute one of NPWT-related mechanisms to accelerate wound healing.

## 1. Introduction

Diabetic foot syndrome (DFS), frequently occurring with diabetic foot ulcers (DFUs), leads to lower extremity amputations and premature death in many patients with diabetes [1–3]. Negative pressure wound therapy (NPWT) has been used as an adjunct treatment for DFUs. Its potential influence on local gene expression and epigenetic methylation in the wound bed has been recently described [4, 5]. However, NPWT's novel mechanism of action may be associated with processes involving the entire organism where signals are transmitted via circulating molecules between the organism's tissues and organs. This intertissue signaling, for example, via circulating microvesicles (MVs), has been linked to the pathomechanism of wound healing [6–8]. MVs represent a heterogeneous population of vesicles that bud off from the plasma membrane and express specific antigens from their parental cells. The main source of circulating MVs originates from different cell populations such as platelets, endothelial cells, neutrophils, and lymphocytes [9–11]. The main function of MVs is to transfer bioactive molecules including proteins, lipids, DNA, mRNA, and miRNA, some of which have important signaling functions [12–14]. Cellular components are selectively recruited into MVs by highly regulated processes [15]. Among these bioactive components, angiogenesis-regulating factors attract the most interest, especially in patients suffering from diabetic complications [16]. MVs contain a variety of factors contributing to their angiogenesis-promoting function, such as angiogenin, vascular endothelial growth factor (VEGF), monocyte chemotactic protein-1 (MCP-1), and also receptor proteins including urokinase-type plasminogen activator receptor (UPAR), receptor-2 for VEGF (VEGF R2), and Tie-2/TEK [17]. Tie-2 belongs to the angiopoietin family of receptors, and its expression is observed mainly on endothelial cells, but also on neutrophils, monocytes/macrophages, and on some smooth muscle cells [11, 18]. Angiopoietin-1 (Ang1) is a proangiogenic factor. Angiopoietin-2 (Ang2), which works through the same receptor as Ang1, was initially described as an antagonist to Ang1 that destabilizes the vasculature [19, 20]. However, recent data suggests that the pro- or antiangiogenic functions of Ang2 depend on the local cytokine milieu [21]. For example, this context-dependent activity of Ang2 is well documented for VEGF. In the presence of VEGF, Ang2 acts more proangiogenic, whereas when VEGF is inhibited, Ang2 acts antiangiogenic [21–24]. Under physiological conditions, the levels of Ang2 are rather low but tend to increase in cancers and inflammatory diseases [20]. In type 2 diabetes (T2DM) patients, levels of circulating Ang2 are increased and associated with chronic complications [25].

The aim of this research was to assess the effect of NPWT on Ang2, a strong modulator of angiogenesis and an inflammation marker, as well as on the circulating plasma levels of its microvesicular receptor Tie2 and the MVs themselves in patients with DFU.

## 2. Materials and Methods

### 2.1. Study Population

Patients were recruited from the Department of Metabolic Diseases' outpatient diabetic foot clinic at the University Hospital in Krakow. We included 69 T2DM patients with neuropathic, nonischemic, and noninfected DFUs. They were assigned either to the standard therapy (ST) group or to the combined standard therapy with NPWT group, with each treatment time lasting 8 ± 1 days. Patients were nonrandomly assigned to the NPWT group but rather chosen based on local and international research and clinical practices, as well as the guidelines concerning NPWT use in DFU, most of which take wound area into account [26, 27]. In short, patients with ulcerations ≤ 1.0 cm^2^ were allocated to the ST group, and the remaining patients were assigned to the NPWT group. Clinical data was obtained from available medical records.

### 2.2. Ethical Approval and Informed Consent

The study protocol was approved by the Jagiellonian University Bioethical Committee and was in accordance with the 1964 Declaration of Helsinki and its later amendments. Patients' written informed consent was obtained prior to inclusion.

### 2.3. Sample Collection and Storage

Blood from the NPWT group was collected in plastic tubes with potassium ethylenediaminetetraacetic acid (EDTA) at an initial (pretreatment) time, and a second (posttreatment) blood draw occurred on day 8 ± 1. In the ST arm, blood samples were also obtained on the same corresponding days (day 0 and day 8). Blood was centrifuged at room temperature for 10 min at 2,700 g with a similar delay (30 min) from the time of blood draw to the time of centrifugation. Then, plasma was separated, stored, and frozen at −80°C until it was assayed.

### 2.4. Laboratory Analysis

Plasma samples were thawed at 37°C in a water bath to avoid cryoprecipitation and centrifuged twice for 15 min at 2,500 g to remove residual platelets. MVs were evaluated by flow cytometry. The average event count was measured, and appropriate dilutions were performed in order to obtain 5,000 events/s. Aliquots of plasma (20 *μ*L) were stained for MVs by incubating with a chosen antibody: Alexa Fluor® 488 anti-human CD202b (Tie2/Tek) Antibody (BioLegend); isotype control Alexa Fluor® 488 Mouse IgG1, *κ* Isotype Ctrl (FC) Antibody (BioLegend); and annexin V (fluorescein isothiocyanate (FITC) annexin V; Annexin V Binding Buffer, BioLegend). After incubating for 30 minutes, samples were then diluted in phosphate-buffered saline to obtain a final volume of 200 *μ*L. Final results were corrected with a dilution factor. All MV analyses were performed on the Apogee A50 micro flow cytometer (Apogee Flow Systems Ltd., Northwood, UK). Every sample was measured for 180 s with a flow rate of 1.5 *μ*L/min; the sample volume was set at 150 *μ*L and the sheath pressure was set at 150 hPa. The trigger was set on a middle angle light scattering (MALS) detector with a voltage of 415 V. For daily calibrations, we used reference beads (ApogeeMix, cat#1493, Apogee Flow Systems Ltd.) composed of a mixture of 180 nm, 240 nm, 300 nm, 590 nm, 880 nm, and 1300 nm silica vesicles with a refractive index of 1.43 and 110 nm and 500 nm green fluorescent (excited by blue laser) latex beads with a refractive index of 1.59. Ang2 evaluations were performed with a Quantikine ELISA Human Angiopoietin-2 Immunoassay (R&D Systems, Minneapolis, USA).

### 2.5. Statistical Analysis

Statistical analysis was performed using Statistica software v. 13.0 (TIBCO Software Inc., CA, USA). The Shapiro-Wilk test was performed to check for a normal distribution of continuous variables. Differences between groups were assessed with the *t*-test or *U* test for normally and nonnormally distributed continuous variables, respectively, and by chi-square or Fischer's exact test for categorical variables. Differences within study groups before and after therapy (day 0 vs. day 8) were checked with the *t*-test or Wilcoxon signed rank test, where applicable. Continuous variables are presented as mean ± SD or median (interquartile range). A *p* value below 0.05 was assumed significant.

## 3. Results

The study group consisted of 69 T2DM patients, 49 of whom were treated with NPWT and 20 treated with ST. The NPWT and ST groups did not differ in basic clinical characteristics, such as age (64.3 ± 10.3 vs. 64.1 ± 6.0 years, *p* = 0.93) and HbA1c levels (51.9 (43.2-58.5) vs. 56.3 (43.7-66.7) mmol/mol, *p* = 0.25). However, they differed by median DFU areas (10.3 (4.2-18.9) vs. 1.3 (0.9-3.4) cm^2^, *p* = 0.0001), which corresponded to the recruitment criteria. The clinical characteristics of the entire study group in which Ang2 was measured and the subgroup in which total MVs, MV Tie+, MV AnnV+, and MV Iso+ were analyzed are presented in Table 1.

At day 0, there were no differences among the examined markers between the NPWT and ST groups. After 8 ± 1 days of therapy (day 8), a significant decrease in the level of Ang2 was observed in the NPWT group, but not in the ST cohort (3.54 (2.40-5.40) vs. 3.32 (2.33-4.61), *p* = 0.02, and 3.19 ± 1.11 vs. 3.19 ± 1.29, *p* = 0.98, respectively). The NPWT therapy did not change the quantity of MVs, MV Tie2+, MV AnnV+, and MV Iso+. Finally, we compared the level of examined particles between the groups at day 8. We found that for total MV quantity, there was a statistically significant difference between the NPWT group and the ST group (12.2∗*E*6 vs. 8.2∗*E*6; *p* = 0.03), while no significant difference was present at day 0 (*p* = 0.07). In addition, there was no difference between the Ang2 and Tie2 plasma levels as well as the MV AnnV+ quantity in this comparison.

Detailed results of the performed measurements are shown in Table 2. The graphical representation of the flow cytometry results is found in the supplementary [Supplementary-material supplementary-material-1].

## 4. Discussion

In this study, we report a potential effect of NPWT on circulating Ang2 and MVs in T2DM patients with noninfected, nonischemic, and neuropathic DFU. Whether it constitutes a novel mechanism of NPWT action requires further research. NPWT's local mechanism of action at the tissue level has been well documented in a recent review [26]. NPWT results in the promotion of wound contraction, tissue granulation, vessel proliferation, neoangiogenesis, epithelialization, and removal of excess extracellular fluid [26]. On the molecular level, NPWT promotes proangiogenic and anti-inflammatory conditions by increasing the expression of growth factors and reducing the expression of inflammatory cytokines [26]. In our previous reports, we saw that NPWT altered the local gene expression involved with wound healing [4]. Moreover, we showed that NPWT's action is mediated through epigenetic alterations resulting mainly in the inhibition of complement system activation [5]. In a very recent study, NPWT increased the number of circulating endothelial progenitor cells in ischemic foot ulcers of diabetic patients which was attributed to the upregulation of systemic and local vascular endothelial growth factors and stromal cell-derived factor-1*α* levels [28]. It should be noted that some unidentified mechanisms might still exist.

In this pilot study, we observed a significant decrease in the level of Ang2 in patients treated with NPWT, but not in the ST group. As mentioned above, since angiopioetin-1 (Ang1) is a proangiogenic factor, Ang2, which acts through the same receptor as Tie2, is thought to be a context-dependent pro- or antiangiogenic factor. In the general population, Ang2 is positively associated with levels of other inflammatory markers, such as hs-CRP and white blood counts [29]. Next, Ang2 has been linked to tumor size and metastatic efficacy, and its inhibition resulted in decreased tumor size and metastatic efficacy [30]. Moreover, Ang2 constitutes a biomarker of cardiovascular risk in individuals with arterial hypertension [31]. Last, but not least, Ang2 is associated with cardiovascular and renal outcomes as well as retinopathy in diabetic patients [32, 33]. Interestingly, recent data suggests that angiopoietin-like protein 2 (ANGPTL2), which is structurally and functionally related to Ang2, was associated with diabetic foot syndrome, and its level correlated with the staging of diabetic foot [34]. Regarding the wound-healing process, it was demonstrated that elevated levels of Ang2 in the absence of VEGF were associated with impaired wound healing, suggesting that a proper balance between Ang2 and VEGF is crucial for maintaining normal wound healing [35]. Our finding of decreased Ang2 levels after NPWT should be considered in the context of other studies showing that NPWT promotes VEGF expression [36]. Moreover, MVs contribute to intercellular EGF-receptor transfer to endothelial cells, which is initiated by VEGF expression [37]. There is also some data on the beneficial effect of Ang2/Tie2 inhibition. For example, Ang2 inhibition prevented transplant ischemia-reperfusion injury and chronic transplant rejection in rat cardiac allografts [38]. In humans, the beneficial effect of Ang2 inhibition was studied mostly in cancer models [20, 30]. On the other hand, Ang-based peptidomimetic compounds resulted in increased granulation tissue and decreased wound closure time in a diabetic mice model [39]. In randomized clinical trials, NPWT proved to be effective in the promotion of wound healing in T2DM patients with diabetic foot syndrome [40, 41]. In light of our pilot study, the beneficial effects of NPWT may be potentially explained by decreased Ang2 levels after NPWT therapy. However, the current study was not designed to directly demonstrate clinical benefits of NPWT on wound healing and the current International Working Group on the Diabetic Foot (IWGDF) guidelines emphasizes the use of NPWT in postoperative rather than in nonsurgical foot wounds [27].

This study has several shortcomings. First of all, it was designed to be nonrandom; thus, it could be biased by several factors including variable ulcer areas in the treatment arms. Second, the sample size was rather low and NPWT use was very brief. Thus, it is unclear whether the negative NPWT findings are true or if they result from the insufficient power to detect the putative impact on anything other than the examined Ang2 circulating molecules. In particular is that these negative findings occurred despite interesting premises; for example, it recently became apparent that MVs are involved during different stages of wound healing, such as coagulation, proliferation, migration, angiogenesis, collagen production, and extracellular matrix remodeling [42]. Moreover, in diabetic rat models, cutaneous wound healing could be accelerated by exosomes from adipose-derived stem cells [43]. Interestingly, in a study on active Charcot neuropathy, it was found that concentrations of extracellular MVs correlated to the elevation of CRP and varying foot temperatures [44]. Such data suggests that MVs can be of great interest as therapeutic agents or as diagnostic tools in patients with complications due to diabetes. Thus, a larger study should be conducted to establish NPWT's effect on the wider spectrum of circulating particles.

## 5. Conclusion

NPWT is observed to influence the level of Ang2 in patients with T2DM who have neuropathic, nonischemic, and noninfected DFUs. Influencing the level of Ang2 may constitute one of NPWT-related mechanisms to accelerate wound healing.

## Figures and Tables

**Table 1:** Clinical characteristics of the study groups.

	Entire group (*n* = 69)			MV subgroup (*n* = 29)		
	NPWT	ST	*p* value	NPWT	ST	*p* value
Sex M/F (*n*)	42/7	15/5	0.31	17/2	8/2	0.59
Age (year)	64.3 ± 10.3	64.1 ± 6.0	0.93	69.2 ± 8.9	62.9 ± 6.6	0.06
BMI (kg/m^2^)	29.6 ± 5.7	30.9 ± 5.1	0.38	27.7 (25.7-30.9)	27.3 (25.6-29.1)	0.91
Duration of diabetes (year)	17.1 ± 8.9	17.1 ± 6.8	0.25	13.4 ± 7.0	16.6 ± 5.6	0.23
Insulin treatment Y/N (*n*)	48/1	18/2	0.20	18/1	9/1	1.00
Duration of insulin therapy (year)	5.0 (1.0-10.0)	6.0 (3.0-15.0)	0.38	4.0 (1.0-8.0)	10.0 (3.0-13.5)	0.10
Daily dose of insulin (unit)	50.0 (40.0-70.0)	50.0 (30.0-60.0)	0.70	43.9 ± 22.5	44.9 ± 27.7	0.96
HbA1c (mmol/mol)	51.9 (43.2-58.5)	56.3 (43.7-66.7)	0.25	47.5 (41.0-56.3)	48.1 (39.9-60.7)	0.96
HbA1c (%)	6.9 (6.1-7.5)	7.3 (6.2-8.3)	0.25	6.5 (5.9-7.3)	6.6 (5.8-7.7)	0.96
eGFR (mL/min/1.73 m^2^)	88.0 (63.0-93.0)	80.5 (58.0-95.5)	0.74	87.0 (55.0-92.0)	76.0 (53.0-98.0)	0.80
Wound area (cm^2^)	10.3 (4.2-18.9)	1.3 (0.9-3.4)	0.0001	12.6 (3.8-19.2)	1.0 (0.8-1.4)	0.001
Duration of the wound (week)	12.2 (10.1-23.7)	10.3 (4.1-18.0)	0.16	12.0 (10.1-22.9)	10.0 (5.1-24.9)	0.30

Data shown as *n* number of cases, mean ± SD (for normally distributed variables) or median and IQR (for nonnormally distributed variables).

**Table 2:** Results of Ang2 and MV marker measurements, before and after 8 ± 1 days of therapy.

							*p* value^1^ (NPWT vs. ST, before therapy)	*p* value^1^ (NPWT vs. ST, after therapy)
	NPWT (*n* = 49)			ST (*n* = 20)				
	Before therapy	After therapy	*p* value	Before therapy	After therapy	*p* value		
Ang2 (ng/mL)	4.38 ± 3.08 3.54 (2.40-5.40)	3.71 ± 1.97 3.32 (2.33-4.61)	0.01^2^	3.19 ± 1.11 3.00 (2.36-4.14)	3.19 ± 1.29 2.91 (2.17-4.32)	0.98^3^	0.21	0.38

	NPWT (*n* = 19)			NPWT (*n* = 10)				
	Before therapy	After therapy	*p* value	Before therapy	After therapy	*p* value		
MV Tie+	4.37 ± 3.19 3.47 (1.80-5.97)	4.88 ± 2.28 4.54 (3.14-7.06)	0.41^3^	7.98 ± 7.28 4.49 (2.79-14.70)	8.31 ± 8.89 4.57 (1.75-12.23)	0.28^2^	0.32	0.98
MV AnnV+	3.01 ± 3.37 1.97 (1.51-3.15)	3.97 ± 2.63 3.91 (1.68-6.33)	0.07^2^	2.38 ± 1.49 2.10 (1.04-3.56)	2.88 ± 2.26 2.16 (1.13-3.79)	0.52^3^	0.51	0.16
MV Iso+	0.90 ± 0.65 0.74 (0.42-1.36)	1.16 ± 0.68 1.17 (0.53-1.84)	0.57^2^	1.04 ± 0.91 0.65 (0.57-1.22)	2.72 ± 4.80 0.74 (0.69-2.26)	0.33^2^	0.73	0.95
Total MVs (10^6^ events/*μ*L)	9.62 ± 13.02 5.12 (3.06-8.89)	8.25 ± 10.83 3.45 (0.94-9.09)	0.55^2^	15.51 ± 10.55 15.26 (4.42-24.24)	12.18 ± 6.00 13.14 (9.30-15.83)	0.26^3^	0.07	0.03

Data shown as mean ± SD and median (IQR). The units of Tie2+, AnnV+, and Iso+ are positive counts per 1000 events. *p* values calculated for ^1^Mann-Whitney test, ^2^Wilcoxon rank test, or ^3^*t*-test.

## Data Availability

The data used to support the findings of this study are available from the corresponding author upon request.

## Supplementary Materials

Supplementary Materials Supplementary Figure 1: flow cytometry analysis—dot plots of Tie2+-, AnnV+-, and Iso+-positive MVs from for ST and NPWT groups at the beginning (initial point of therapy, IT) and after therapy (AT).
