# Supplementary material for: Effects of Negative Pressure Wound Therapy on Levels of Angiopoetin-2 and Other Selected Circulating Signaling Molecules in Patients with Diabetic Foot Ulcer
Source: J Diabetes Res. 2019 Oct 28;2019:1756798. doi: 10.1155/2019/1756798 (PMC6855047; doi:10.1155/2019/1756798)
Supplement: Supplementary Materials — Supplementary Figure 1: flow cytometry analysis—dot plots of Tie2+-, AnnV+-, and Iso+-positive MVs from for ST and NPWT groups at the beginning (initial point of therapy, IT) and after therapy (AT). [file 1756798.f1.docx]

Supplementary Figure 1. Flow cytometry analysis - dot plots of Tie2+, AnnV+ and Iso+ positive MVs from for ST and NPWT groups at the beginning (Initial Point of Therapy – IT) and after therapy (AT).

| MVs_ST_IT | MVs_ ST_AT | MVs_NPWT_IT | MVs_NPWT_AT |
| --- | --- | --- | --- |
| 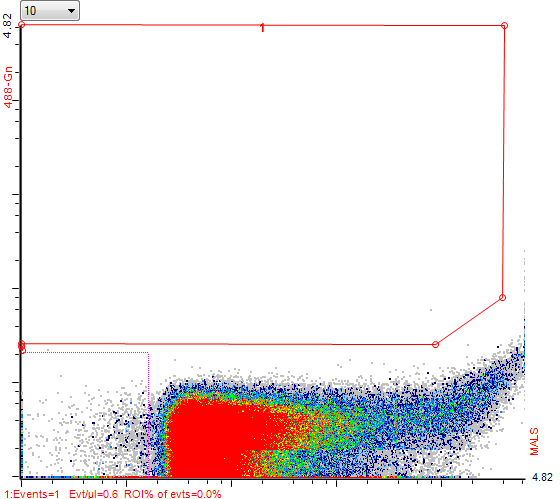 | 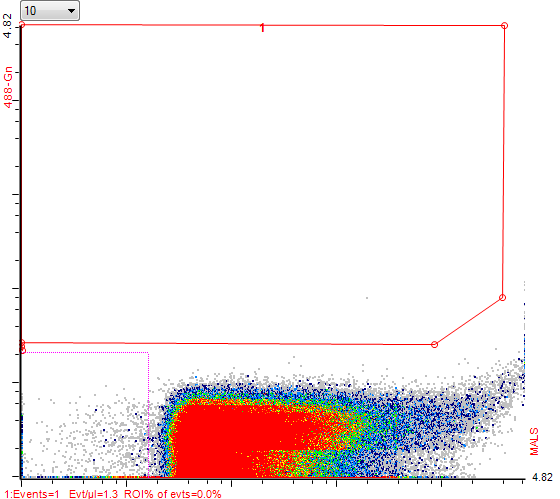 | 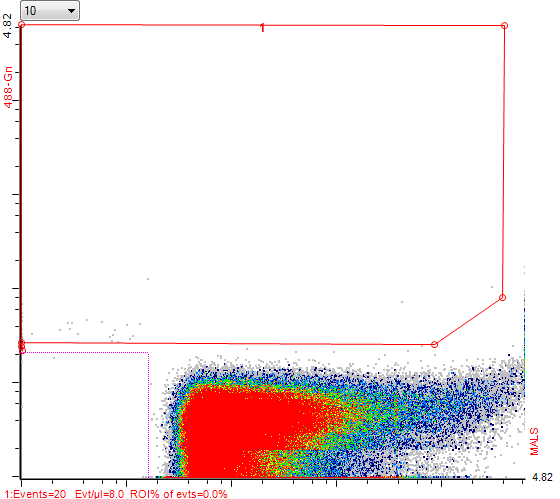 | 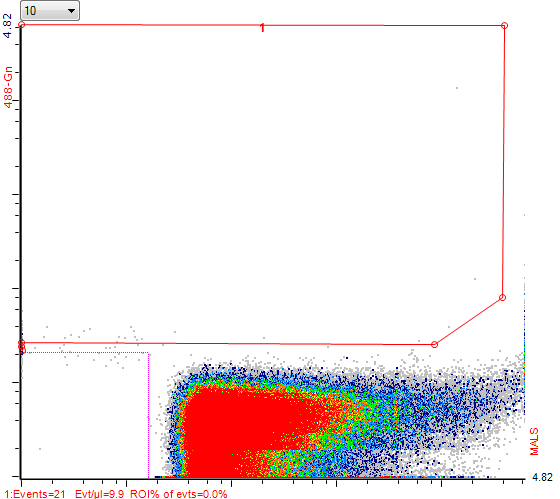 |
| AnnV^+^_ ST_IT | AnnV^+^_ ST_AT | AnnV^+^_ NPWT_IT | AnnV^+^_ NPWT_AT |
| 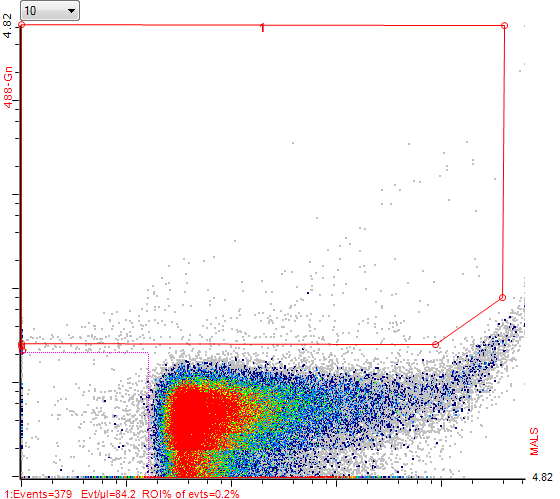 | 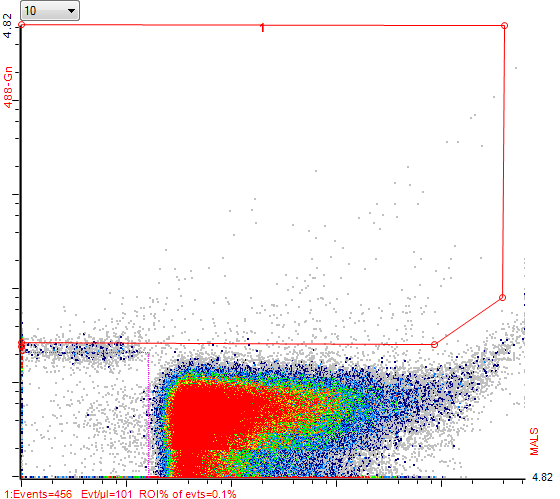 | 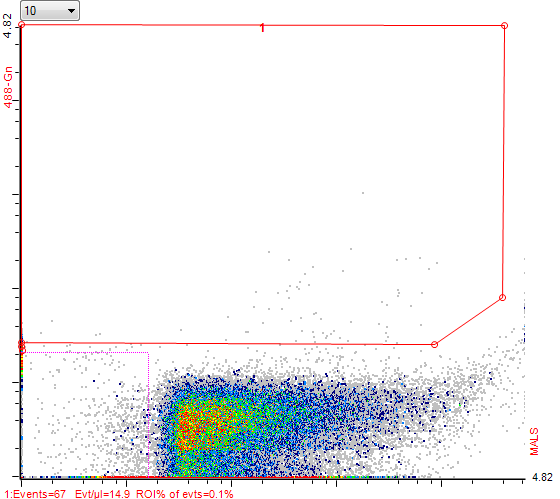 | 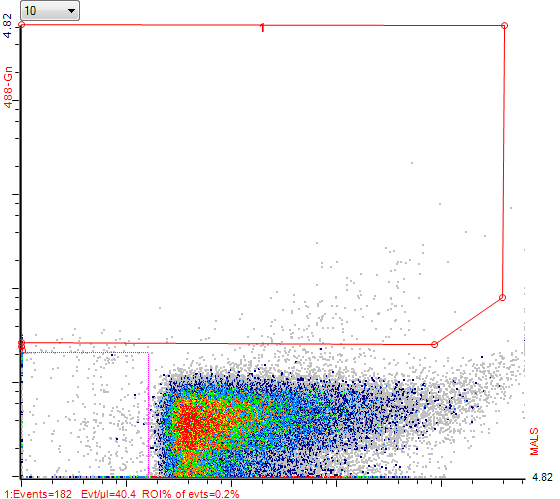 |
| Tie2^+^_ ST_IT | Tie2^+^_ ST_AT | Tie2^+^_ NPWT_IT | Tie2^+^_ NPWT_AT |
| 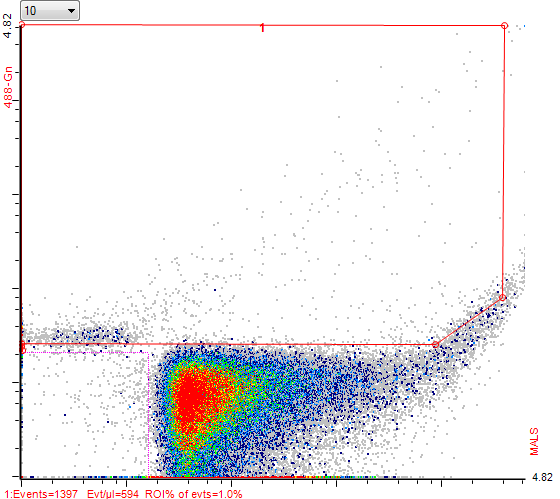 | 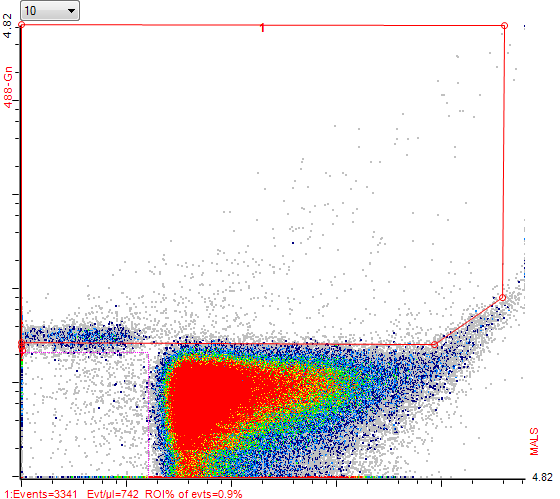 | 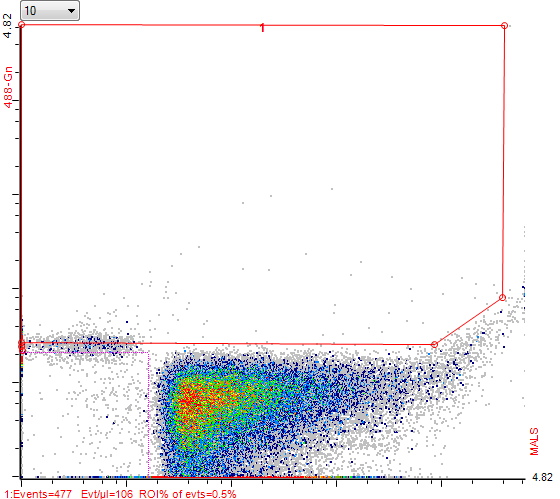 | 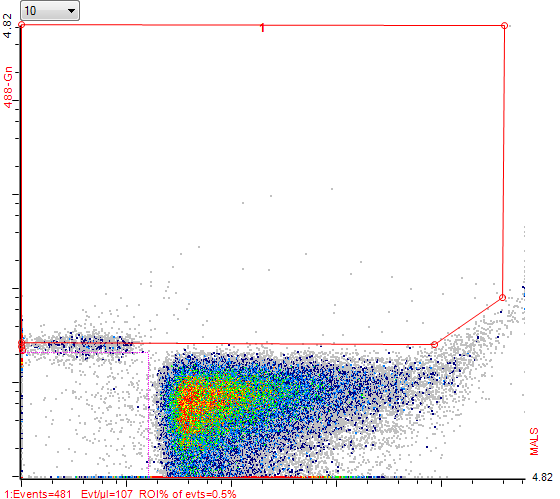 |
| Iso^+^_ ST_IT | Iso^+^_ ST_AT | Iso^+^_ NPWT_IT | Iso^+^_ NPWT_AT |
| 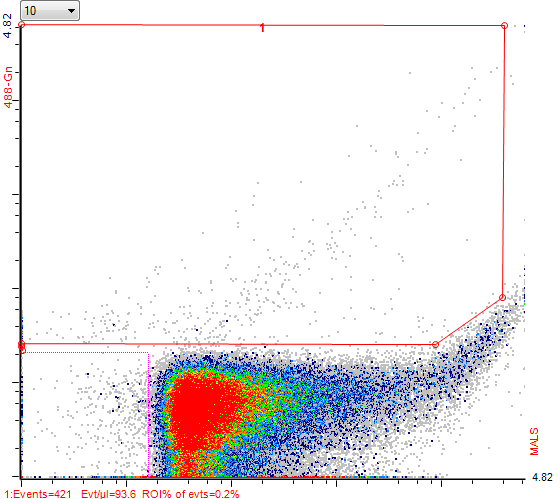 | 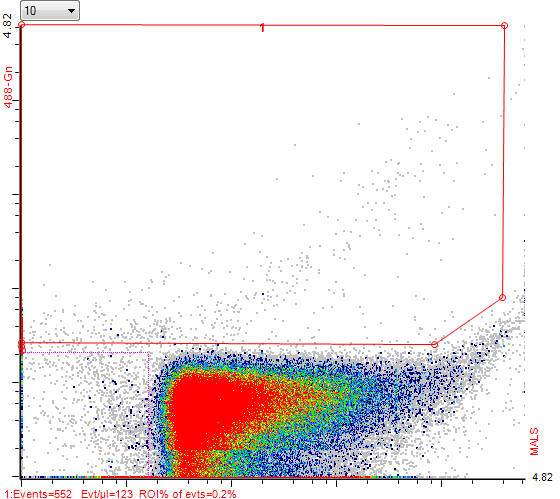 | 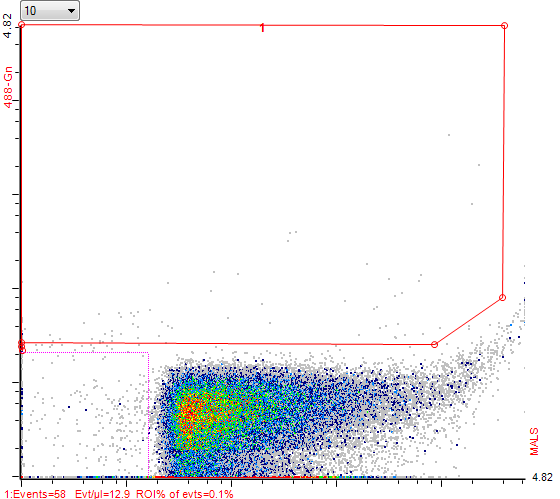 | 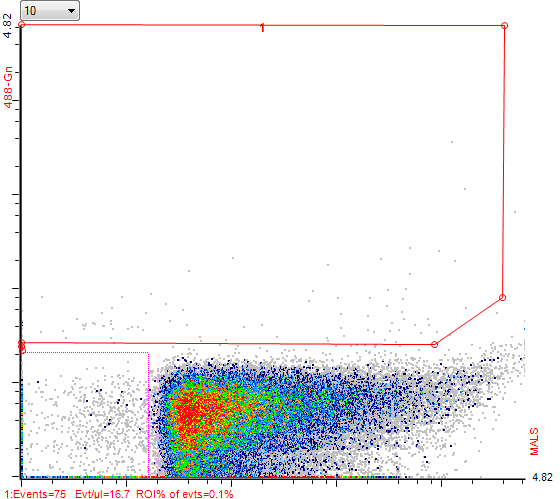 |
